# The Multiple Roles of LCCL Domain-Containing Proteins for Malaria Parasite Transmission

**DOI:** 10.3390/microorganisms12020279

**Published:** 2024-01-29

**Authors:** Sandra Bennink, Gabriele Pradel

**Affiliations:** Division of Cellular and Applied Infection Biology, Institute of Zoology, RWTH Aachen University, Worringerweg 1, 52074 Aachen, Germany; bennink@bio2.rwth-aachen.de

**Keywords:** malaria, *Plasmodium*, protein–protein interaction, transmission, multi-protein complex, gametocyte, oocyst, sporulation, sporozoite, crystalloid

## Abstract

Multi-protein complexes are crucial for various essential biological processes of the malaria parasite *Plasmodium*, such as protein synthesis, host cell invasion and adhesion. Especially during the sexual phase of the parasite, which takes place in the midgut of the mosquito vector, protein complexes are required for fertilization, sporulation and ultimately for the successful transmission of the parasite. Among the most noticeable protein complexes of the transmission stages are the ones formed by the LCCL domain-containing protein family that play critical roles in the generation of infective sporozoites. The six members of this protein family are characterized by numerous adhesive modules and domains typically found in secreted proteins. This review summarizes the findings of expression and functional studies on the LCCL domain-containing proteins of the human pathogenic *P. falciparum* and the rodent-infecting *P. berghei* and discusses the common features and differences of the homologous proteins.

## 1. Introduction

The tropical disease malaria stands out as a significant global public health challenge, exerting a substantial impact on numerous nations worldwide. Particularly vulnerable are young children and pregnant women. Approximately 50% of the global population resides in regions, spanning 87 countries and territories, where the risk of malaria transmission is prevalent. In the year 2020, this disease led to around 241 million clinical episodes and resulted in 627,000 fatalities. Notably, an estimated 95% of these deaths occurred in Africa [1].

Malaria is caused by infections with protozoan parasites of the genus *Plasmodium* that are transmitted between humans by blood-feeding *Anopheles* mosquitoes. After being injected into the human, the parasites quickly reach the liver and replicate asexually inside hepatocytes during an asymptomatic infection phase. Once released from the hepatocytes, the parasites actively invade erythrocytes, thereby starting the erythrocytic replication cycle, during which they multiply inside erythrocytes and subsequently invade new red blood cells. The repeating burst of erythrocytes is responsible for symptoms like fever and anemia and, in severe cases, can lead to organ failure and death (reviewed in [2]).

A proportion of the red-blood-cell-infecting parasites enter the sexual pathway to form sexual precursor cells, the gametocytes, and this process is enforced by external stress factors such as high parasitemia, drug pressure or the lack of select lipid precursors. In the human malaria parasite *P. falciparum*, the development of intraerythrocytic gametocytes spans approximately 10 days, during which the gametocytes are sequestered in the bone marrow. Following maturation, the gametocytes circulate in the peripheral blood system to be taken up by a mosquito during a bite (reviewed in [3,4]).

Once ingested with the blood meal, the gametocytes become activated by the environmental factors of the mosquito’s midgut like a drop in temperature and the presence of the mosquito-derived molecule xanthurenic acid and undergo gametogenesis. The sexual phase of the parasite within the mosquito’s gut persists for around 20 h and involves the rapid conversion of activated gametocytes into fertile male and female gametes, which necessitates their release from the enclosing erythrocyte, as well as a second slower conversion of the resulting zygote into a mobile and invasive ookinete. The ookinete promptly exits the gut lumen by traversing the midgut epithelial cell layer, settles at the basal site of the midgut epithelium and transforms into a sessile oocyst that undergoes sporogonic replication. After approximately two weeks, the newly formed infective sporozoites egress from the oocyst and migrate through the hemolymph to the salivary glands of the mosquito, after which they will be transmitted to the new human host with the mosquito’s next blood meal (reviewed in [3,5]).

A fascinating family of proteins has been shown to be crucially involved in the transmission of *Plasmodium* parasites, especially in the development of infective sporozoites. These proteins share a consensus LCCL domain and are highly conserved among apicomplexan parasites with homologous proteins of the human malaria parasite *P. falciparum* and the rodent-infecting *P. berghei*, exhibiting similar domain architectures. The protein family is synthetized during the early gametocyte stage until oocyst maturation, and while the subcellular localization differs significantly between the two malaria species, loss-of-function mutations result in comparable phenotypes. In this review, we discuss the common features and differences of LCCL domain-containing proteins in *P. falciparum* and *P. berghei*, with a special focus on their role during the transmission of the parasites to the mosquito vector.

## 2. Structure and Origin of LCCL Modules

The LCCL module is composed of approximately 100 amino acids containing four highly conserved cysteine residues that presumably form two disulfide bonds. The C-terminal region further contains a highly conserved histidine residue that is embedded in the conserved motif YxxxSxxCxAAVHxGVI. The LCCL module has been identified in proteins of various metazoans, ranging from protists to humans. Structure predictions and modulations suggest that the LCCL module is arranged into a unique fold comprising a centrally located α-helix surrounded by two β-sheets (Pfam entry PF03815; InterPro entry IPR004043). It has been proposed that the LCCL module is an autonomously folding domain that was evolutionary spread by exon-shuffling and hence was involved in the construction of various modular proteins. It is often found in extracellular proteins in association with other adhesion domains like C-type lectin domains, von Willebrand type A domains and discoidin lectin domains [6].

The LCCL domain is named after the three proteins in which it was first identified, i.e., the horseshoe crab *Limulus* clotting factor C, the cochlear protein Coch-5b2 and the late gestation lung protein Lgl1. The *Limulus* clotting factor C is a serine protease that initiates a coagulation cascade in response to Gram-negative bacteria being present in the arthropod’s hemolymph as a means of an antibody-independent immune reaction. In addition to the LCCL domain, the *Limulus* clotting factor C contains five complement control protein domains, one epidermal growth factor-like domain, one C-type lectin like domain and a C-terminally located serine protease domain [7,8,9].

Coch-5b2, also named cochlin, is a highly abundant extracellular matrix protein in the human cochlea which contains an N-terminal LCCL domain followed by two von Willebrand type A (vWA) domains [6,10]. During bacterial infections of the cochlea, the LCCL domain is enzymatically cleaved and secreted into the perilymph. Here, it inhibits bacteria from further spreading and activates innate immunity by recruiting neutrophils and macrophages, thereby protecting essential auditory structures ([11], reviewed in [12]). Interestingly, a range of mutations either in the LCCL domain or the vWA domains of cochlin are associated with hearing loss through the misfolding or secretion failure of the protein [13], and hearing loss in a cochlin knockout mouse model can be treated by medication with LCCL peptides [11]. Recently it was described that the LCCL peptide from cochlin is also cleaved and secreted in the cochlea after noise exposure, thereby aggravating noise-induced hearing loss [14]. In addition to its role in the inner ear, cochlin is further expressed by follicular dendritic cells and found in the spleen and lymph nodes, where it promotes antibacterial immunity via the regulation of cytokine production and the recruitment of immune effector cells [15].

Finally, Lgl1 is important for human fetal lung development through modulating proliferation, apoptosis and migration of fibroblasts [16]. In addition to two LCCL domains, it exhibits an N-terminal SCP (sperm-coating protein) domain and was shown to bind to lipid A of lipopolysaccharides (LPS), the major immunostimulatory component of the outer membrane of Gram-negative bacteria [17,18].

Although these three LCCL domain-containing proteins are phylogenetically unrelated, they all bear signal peptides and are found in the extracellular space. For all of these proteins, interactions with bacterial cell wall components are described, suggesting their participation in antimicrobial host defense mechanisms through the recognition of cell surface carbohydrates.

## 3. Architecture of the Plasmodial LCCL Domain-Containing Proteins

LCCL domain-containing proteins are highly conserved among the apicomplexan clade. Orthologous proteins containing LCCL modules have been identified in *Plasmodium, Cryptosporidium, Toxoplasma, Babesia* and *Theileria*, while LCCL domain-containing proteins were not found in any other protozoan organism, pointing to an apicomplexan-specific function of these proteins [19,20,21,22,23,24,25]. Considering the anticipated role of the LCCL module in microbial binding during host cell defense (see above), lateral gene transfer as a potential origin of the corresponding genes has been suggested. In accord with this hypothesis, the genomes of apicomplexan parasites carry a large repertoire of genes of plant, animal and bacterial origins and further contain remnants of retroviral and transposable elements (reviewed in [26,27,28]).

**Figure 1:**
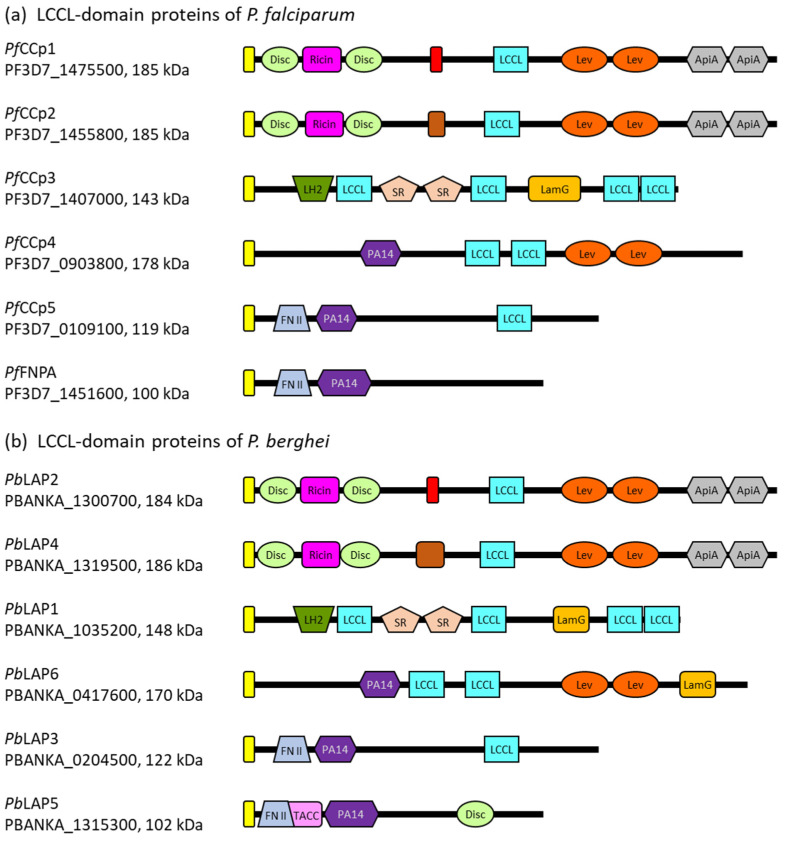
The domain architecture of the LCCL domain-containing proteins. (**a**) Domain structure of the *Pf*CCp proteins of *P. falciparum*. (**b**) Domain structure of the *Pb*LAP proteins of *P. berghei*. The gene IDs according to PlasmoDB [32] and molecular weights are indicated. Yellow box, signal peptide; light-green box, discoidin domain; pink box, ricin domain; red box, fibrinogen-related domain; light-blue box, LCCL domain; orange box, levanase domain; grey box, ApiA domain; brown box, COLFI domain (fibrillar collagen C-terminal domain); dark-green box, LH2 domain (lipoxygenase homology 2 domain); light-brown box, SR domain (scavenger receptor-like domain); light-orange box, LamG domain (Laminin G-like domain); dark-purple box, PA14 domain (anthrax protective antigen domain); lavender box, fibronectin type II domain; pale-rose box; TACC domain (transforming acidic coiled-coil-containing protein domain). The domain architecture predictions are based on the NLM’s Conserved Domain Database [33] and previous studies [21,27,34].

*Plasmodium* parasites express five LCCL domain-containing proteins of molecular weights between ~120 kDa and 185 kDa. In addition to the characteristic LCCL module, the five proteins comprise various other domains with adhesive properties, including domains with carbohydrate- or protein-binding motifs. The LCCL domain-containing proteins further share a predicted signal peptide, indicating an extracellular location, but lack transmembrane domains or GPI anchor signal sequences (Figure 1). In *P. falciparum*, the five LCCL domain-containing proteins are also called *Pf*CCp1 to *Pf*CCp5 (LCCL domain-containing protein), while in the rodent malaria parasite *P. berghei*, the homologs are termed *Pb*LAP (LCCL-lectin adhesive-like proteins). The sequences of the genes encoding these proteins have been analyzed and revised multiple times during the last two decades, resulting in variations of the adhesion domain architectures, as discussed below.

The *Pf*CCp1 and *Pf*CCp2 proteins of *P. falciparum* are paralogs that arose through gene duplication. The *P. berghei* orthologs of *Pf*CCp1 and *Pf*CCp2 are named *Pb*LAP2 and *Pb*LAP4, respectively, showing 67% and 66% sequence identity. The four proteins share similar domain architectures, with the single LCCL domain being located in the central part of each protein. In their N-terminal parts, the proteins possess a ricin domain flanked on both sides by discoidin/F5-F8 type-C domains, which have assigned roles in carbohydrate and phospholipid binding, respectively [29,30,31]. These domains are followed by a predicted fibrinogen-related/fibrillar collagen C-terminal domain, previously also named the NEC domain, which exhibits carbohydrate-binding properties and is adjacent to the LCCL domain [21]. Previous studies have additionally predicted two levanase domains with assigned carbohydrate-binding functions followed by two apicomplexan-specific cysteine-rich domains termed ApiA that are located C-terminally to the LCCL domain of *Pf*CCp1 and *Pb*LAP2 as well as *Pf*CCp2 and *Pb*LAP4 [21,22,27].

*Pf*CCp3 and its ortholog *Pb*LAP1 share 63% of their sequence identity and an identical architecture comprised of multiple defined adhesion domains. In the N-terminal region, the protein sequences exhibit an LH2 (lipoxygenase homology 2) domain, which is found in several membrane-associated proteins and thought to mediate membrane attachment via protein binding partners (reviewed in [35]). The LH2 domain is followed by the first out of four LCCL domains. In the central regions of the proteins, a tandem repeat of a scavenger receptor-like (SR) domain is present. This cysteine- and disulfide-rich domain of extracellular proteins is believed to be involved in protein–protein interactions and also interacts with other SR domains ([36], reviewed in [37]). The second LCCL domain is located between the SR-repeat and a Laminin G-like domain (LamG), which is typically involved in processes like adhesion or migration [38]. It has been further predicted to resemble the structure of the pentraxin domain, which is able to interact with other pentraxin domains as well as complement control protein modules and which plays a role in lipoprotein and carbohydrate binding during immune defense ([39], reviewed in [40]). Two more LCCL domains are situated at the very C-terminal part of *Pf*CCp3 and *Pb*LAP1.

*Pf*CCp4 and its ortholog *Pb*LAP6 as well as *Pf*CCp5 and *Pb*LAP3 have less striking adhesion domain architectures than the above described LCCL domain-containing proteins. While *Pf*CCp4 and *Pb*LAP6 share 47% of their identity, 51% of sequence identity is found between *Pf*CCp5 and *Pb*LAP3. Although previous predictions assigned two LCCL domains to *Pf*CCp4 and *Pb*LAP6 [34,41], newer annotations only predict one central LCCL domain in the two protein sequences. *Pf*CCp5 and *Pb*LAP3 also comprise a single LCCL domain, which is found at the C-terminal portion of the proteins. The four proteins further share an N-terminal PA14/anthrax protective antigen domain, which is suggested to have carbohydrate-binding functions [42]. Previous studies also described two C-terminal levanase domains for *Pf*CCp4 and *Pb*LAP6, a C-terminal LamG domain for *Pb*LAP6 and an N-terminal fibronectin type II domain for *Pf*CCp5 and *Pb*LAP3 [21,27].

A sixth member has been assigned to the LCCL domain-containing families of *P. falciparum* and *P. berghei* due to the structural similarity of this protein with *Pf*CCp5 and *Pb*LAP3, respectively, although it lacks an LCCL domain. The protein is referred to as *Pf*FNPA (fibronectin type II and PA14 domain protein) in *P. falciparum* and *Pb*LAP5 in *P. berghei*, and the two orthologous proteins share 67% of their sequence identity. In addition to the PA14/anthrax protective antigen domain, *Pb*LAP5 contains a predicted N-terminal TACC (transforming acidic coiled-coil-containing protein) domain, which is usually found in centrosomal proteins [43]. Previously, a (name-giving) N-terminal fibronectin type II domain was assigned to both *Pf*FNPA and *Pb*LAP5, and a C-terminal discoidin domain was described for *Pb*LAP5 [21,27].

## 4. Expression of the Plasmodial LCCL Domain-Containing Proteins in the Transmission Stages

### 4.1. LCCL Domain-Containing Protein Complexes in P. falciparum Gametocytes

Several studies have investigated the plasmodial LCCL domain-containing proteins on the transcript and protein levels. In *P. falciparum*, the expression of the *Pf*CCp proteins is specific for the gametocyte stages [20,21,44,45]. While *Pf*CCp4 is detectable as early as stage I gametocytes, all other *Pf*CCp members are found in gametocyte stages II–V. The *Pf*CCp proteins are mainly expressed in the female gametocytes [45,46]. In accord with the predicted signal peptide, the proteins follow the secretory pathway and are released into the lumen of the parasitophorous vacuole, where they associate with the plasma membrane of the gametocyte [21,45,47]. While *Pf*CCp1 to *Pf*CCp4 are evenly distributed in the vacuolar space, *Pf*CCp5 and *Pf*FNPA localize predominantly at the poles of the gametocytes [45]. During gametogenesis, *Pf*CCp4 in particular remains detectable on the surface of emerged macrogametes, but the expression of the *Pf*CCp proteins ceases a few hours later [21,45].

**Figure 2:**
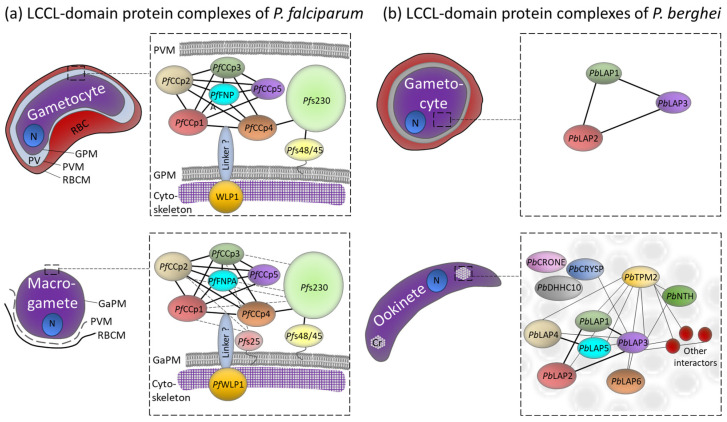
Complex formation and subcellular localization of the LCCL-domain protein complexes. (**a**) *Pf*CCp-based complexes in the gametocytes and macrogametes of *P. falciparum*. (**b**) *Pb*LAP-based complexes in gametocytes and ookinete crystalloids of *P. berghei*. The proposed protein networks are shown, and their rearrangements during the sexual phases of the parasites are indicated. Cr, Crystalloid; GaPM, gamete plasma membrane; GPM, gametocyte plasma membrane; N, nucleus; PV, parasitophorous vacuole; PVM, PV membrane; RBC, red blood cell; RBCM, RBC membrane. Predicted interactions are proposed based on co-immunoprecipitations followed by Western blot analysis or mass spectrometry [34,48,53]. *P. falciparum*: Dashed lines indicate further interactions strengthening the complex after gametogenesis. *P. berghei*: Thin lines indicate weaker interactions that are only detectable after crosslinking. Modified from [49].

Protein–protein interaction studies revealed that the *Pf*CCp proteins assemble to multi-protein complexes via their adhesive domains (Figure 2a). Particularly, the LCCL and the SR domains are involved in these interactions [48]. The multi-protein complexes comprise additional adhesion proteins like *Pf*s230, which is linked to the complex via its interaction with *Pf*CCp4 [45,49]. *Pf*s230 is a member of the 6-cys protein family; the protein is associated with the plasma membrane of the gametocyte and later exposed on the gamete’s surface, where it mediates the binding of microgametes to uninfected red blood cells during exflagellation [50]. *Pf*s230 interacts with the GPI-anchored 6-cysteine protein *Pf*s48/45, and this interaction couples the whole *Pf*CCp-based protein complex to the gametocyte’s plasma membrane [49,51,52]. The stability of the *Pf*CCp-based complex requires the presence of all family members, since the lack of one protein results in the complete or partial loss of the other *Pf*CCp proteins. The presence of *Pf*s230, however, is not affected by the loss of *Pf*CCp proteins [45,47,48,49].

Following gametogenesis, the *Pf*CCp-based complex is additionally stabilized by the GPI-anchored protein *Pf*s25. In non-activated gametocytes, transcripts encoding *Pf*s25 are translationally repressed via ribonucleoprotein particles, but the repression is released upon gametocyte activation (reviewed in [3,54]). Once present on the macrogamete’s surface, *Pf*s25 interacts with select *Pf*CCp proteins [49] (Figure 2a). At the same time, *Pf*s230 is processed, resulting in its enhanced interaction with the multi-protein complex. Both the incorrect processing of *Pf*s230 and its absence lead to the proteolysis of the *Pf*CCp proteins and their release from the multi-protein complex [49,55]. Cell binding assays, using recombinant *Pf*CCp peptide-coated fluorescent latex beads, demonstrated the binding of the beads to the surface of newly emerged macrogametes but not to non-activated gametocytes due to specific interactions of the recombinant peptides with endogenous *Pf*CCp proteins that are exposed on the macrogamete surface [48].

Recently the WD40-repeat protein *Pf*WLP1 has been described as a component of the *Pf*CCp-based protein complex as it co-immunoprecipitates with *Pf*CCp1 and *Pf*s230 in non-activated and activated gametocytes. *Pf*WLP1 is an intracellular protein that accumulates underneath the gametocyte´s plasma membrane [56]. Since the knockdown of *Pf*WLP1 results in reduced protein levels of *Pf*CCp1 and *Pf*CCp2, it is speculated that *Pf*WLP1 ensures the stability of the *Pf*CCp-complex [57] (Figure 2a). It is postulated that the cross-membrane interaction of the intracellular *Pf*WLP1 with the extracellular protein complexes is mediated via an unknown membrane-spanning linker protein. One possible candidate is the transmembrane protein MTRAP, an integral component of the plasma membrane, which is essential for the rupture of the parasitophorous vacuole during the egress of the activated gametocytes from the enveloping red blood cell ([58,59,60], reviewed in [61]). It has been suggested that MTRAP connects vacuolar components with the intracellular cytoskeleton, presumably via *Pf*WLP1-binding, thereby bridging the plasma membrane of the gametocytes with the parasitophorous vacuole membrane, and that this linkage has to be dismantled during egress [57].

### 4.2. Aggregation of the LCCL Domain-Containing Proteins in the Transmission Stages of P. berghei

Transcript analysis revealed that the *P. berghei* LAP proteins are predominantly expressed in the transmission stages, i.e., gametocytes and ookinetes [20,62,63] (Figure 2b). Interestingly, for *Pb*LAP4 to *Pb*LAP6, protein expression was not detected in the gametocyte stages, although transcript levels were high, indicating that translational repression regulates the presence of these *Pb*LAP proteins [63]. Sex-specific proteomics studies suggest that *Pb*LAP1 to *Pb*LAP3 are exclusively expressed in female gametocytes [64]. The sex specificity has been confirmed by genetic cross studies using either male- or female-deficient parasites, which revealed that the functional *pblap* genes are inherited from the females [65]. In the gametocytes, *Pb*LAP1 to *Pb*LAP3 are located in cytosolic granules [66,67], whereas in the ookinete stage, all six *Pb*LAP proteins are located at two or three focal spots in the cell, often close to clusters of malaria pigment [63,66,67]. Early studies have further reported *Pb*LAP1 expression in sporozoites [19,24]; however, later immunofluorescence studies were not able to confirm the presence of *Pb*LAP proteins in these stages [66]. Unlike *Pf*CCp proteins, *Pb*LAP proteins are not co-dependently expressed. Nevertheless, they appear to have a conformational interdependence, wherein the absence of one *Pb*LAP protein leads to the misfolding of other *Pb*LAPs [68].

The distinct *Pb*LAP-containing spots in the ookinetes were later identified as crystalloids, which are transient organelles of ookinetes and young oocysts found in rodent malaria parasites. The crystalloids are aggregations of tightly packed spherical particles, which are usually surrounded by hemozoin-containing vacuoles and whose assembly relies on microtubule-based endoplasmic-reticulum-derived vesicle transport as shown via inhibitor studies ([69], reviewed in [70,71]). In the majority of ookinetes, initially, two crystalloids are present, which presumably fuse during oocyst transition, resulting in a single large crystalloid in the oocyst [69]. *Pb*LAP1 to *Pb*LAP3 form an initiator protein complex in the gametocyte stages, which relocates to the crystalloids during ookinete development [34,66,67,68]. After fertilization, the synthesis of translationally repressed *Pb*LAP4 to *Pb*LAP6 is initiated, and the proteins are then also recruited to the crystalloid-resident *Pb*LAP complex; this process is apparently facilitated through the SR domains of *Pb*LAP1 [34]. The crystalloid formation further seems to be dependent on the presence of the functional *Pb*LAP complex, since mutations of *Pb*LAP proteins lead to abnormal crystalloid development that ultimately results in a phenotype of size-reduced oocysts and premature sporulation (see below) [69,72].

In addition to the *Pb*LAP complex, several more crystalloid-associated proteins have been identified, such as CRYSP and CRONE which were named after their phenotypes “crystalloid needed for sporozoites” and “crystalloid oocyst not evolving”, respectively. While *crone* gene deletion mutants exhibit defective sporozoite formation and budding, *crysp* gene deletion mutants are capable of forming sporozoites; however, they fail to infect the salivary glands [73,74]. Further, the S-acyl-transferase DHHC10 has been shown to localize to the crystalloids and to be required for the biogenesis of the organelle [75]. In line with these results, pull-down experiments revealed that the *Pb*LAP complex is part of a larger protein network comprising approximately 50 crystalloid proteins. Among them, the NAD(P) transhydrogenase NTH, members of the CPW-WPC domain-containing protein family and a TPM domain-containing membrane protein called TPM2 have been identified [53] (Figure 2b).

## 5. The Role of the Plasmodial LCCL Domain-Containing Proteins during Malaria Transmission

In attempts to elucidate the functions of plasmodial LCCL domain-containing proteins, loss-of-function studies have been performed on *P. falciparum* and *P. berghei* using mutants lacking or exhibiting truncated versions of the respective gene. Even though for *P. falciparum*, the transcript and protein expression of the *Pf*CCp proteins peak at the gametocyte stage, none of the investigated *Pf*CCp-deficient lines displayed a phenotype in gametocyte development or during exflagellation. Furthermore, the lines did not show defects in ookinete and oocyst formation nor in sporulation [21,45,48]. However, when *Pf*CCp2- or *Pf*CCp3-deficient gametocytes were fed to *Anopheles* mosquitoes via membrane feeding, no sporozoites were detected within or associated with the salivary glands, nor within the hemocoel of the mosquitoes, indicating that *Pf*CCp2 and *Pf*CCp3 are essential for the sporozoite transition from the midgut to the salivary glands [21]. Interestingly, when the expression of the *Pf*CCp-complex stabilizer *Pf*WLP1 is knocked down, exflagellation is severely impaired compared to wildtype parasites [57]. These data suggest that the loss of *Pf*WLP1 not only affects *Pf*CCp-complex stability but also other cellular processes of gametogenesis, like cytoskeletal reassembly during exflagellation. Furthermore, antibodies directed against the *Pf*CCp proteins are capable of inhibiting exflagellation in active serum. This effect, however, is not measurable in heat-inactivated serum, pointing to an involvement of an active complement in the process [45].

The loss-of-function phenotype of the *Pb*LAP proteins of *P. berghei* also manifests in the oocyst stage. When mosquitoes are fed with mouse blood containing *Pb*LAP-deficient gametocytes, ookinetes develop normally, and the numbers of oocysts are comparable to the wildtype; however, the oocysts appear degenerated, or their diameters are significantly enlarged compared to the control [19,65,66,69,76]. Because parasites lacking *Pb*LAP1 (also termed *Pb*SR in this study) are able to sporulate in *in vitro* assays, it was proposed that the loss-of-function phenotype involves mosquito factors [66]. Double-knockout mutants lacking *Pb*LAP1 and *Pb*LAP2 or *Pb*LAP2 and *Pb*LAP6 display the same phenotype as single-knockout mutants, i.e., aborted sporozoite formation [62]. Interestingly, fluorophore-tagged *Pb*LAP4 mutants exhibit smaller oocysts than control parasites, and the number of salivary glands in sporozoites is reduced, indicating that sporozoite egress or survival is compromised [72]. The tagging of *Pb*LAP1 on the other hand, did not affect the functionality of the protein and resulted in normal sporulation and transmission [66]. Recently, it has been shown that in *Pb*LAP1-deficient parasites, the gene expression of major sporozoite genes is dysregulated, which might explain impaired sporulation in the absence of *Pb*LAP proteins [77]. A significant downregulation during early oocyst development was observed for genes encoding important sporozoite transcription factors (Ap2-SP [78] and Ap2-SP2 [79]) as well as proteins of cytokinesis (i.e., CSP [80] and IMC1a [81,82]) or other sporozoite functions (i.e., TRAP [83,84] and SPECT2 [85]).

Furthermore, crystalloid assembly is affected in ookinetes lacking *Pb*LAP proteins. In the absence of *Pb*LAP1 or *Pb*LAP3, no crystalloids are formed in the ookinetes, whereas the deletion of the LCCL domain of *Pb*LAP3 or the C-terminal GFP-tagging of *Pb*LAP4 results in an abnormal crystalloid morphology [66,69,72]. These data suggest that the *Pb*LAP proteins are indirectly involved in sporogony, likely by participating in crystalloid biogenesis. Supporting the functional link between crystalloids and sporogony, mutants deficient in the crystalloid proteins DHHC10 and NTH fail to assemble crystalloids and to sporulate [75,86].

## 6. Conclusions

In recent years, LCCL domain-containing proteins have been suggested as playing a major role in malaria transmission. A shared feature of the LCCL domain-containing proteins of *P. falciparum* and *P. berghei* is the presence of multiple adhesive domains, which are important for protein–protein and protein–carbohydrate interactions, and indeed, the proteins assemble into complexes via their adhesion domains to form extensive protein networks. However, although the loss-of-function phenotypes of these proteins are comparable in that their deficiency mutants do not form infective sporozoites, the exact roles of the proteins between both species seem to differ quite substantially. In *P. falciparum*, the *Pf*CCp proteins interact with other extracellular proteins to form multi-protein complexes that are linked to the plasma membrane of gametocytes, and later, the complexes facilitate the adhesiveness of the emerging gametes. In *P. berghei*, while *Pb*LAP expression also starts in gametocytes, the proteins are not secreted but rather locate to the intracellular crystalloids of ookinetes.

The LCCL proteins were one of the first protein families identified and characterized following the sequencing of the *P. falciparum* and *P. berghei* genomes at the beginning of this millennium. The large number of adhesion domains, which were either acquired through horizontal gene transfer or are specific to the Apicomplexa, originally made the proteins very attractive vaccine candidates. However, due to the unexplained delayed phenotype that manifests during sporozoite maturation, the proteins lost their charm as transmission-blocking vaccine targets, and in consequence, interest in the LCCL domain-containing proteins has waned in recent years. Thus, despite twenty years of research, the function of these fascinating adhesion proteins in the transmission stages remains unclear. Given the difficulty of the technical setup required for studies on malaria-infected mosquitoes and the dwindling third-party funding for basic research, it remains to be seen whether the functions of LCCL domain-containing proteins will ever be sufficiently deciphered.

## Data Availability

No new data were created or analyzed in this study. Data sharing is not applicable to this article.

## References

[1] WHO (World Health Organisation) (2022). World Malaria Report 2022.

[2] Poespoprodjo J.R., Douglas N.M., Ansong D., Kho S., Anstey N.M. (2023). Malaria. Lancet.

[3] Bennink S., Kiesow M.J., Pradel G. (2016). The Development of Malaria Parasites in the Mosquito Midgut. Cell. Microbiol..

[4] Josling G.A., Williamson K.C., Llinás M. (2018). Regulation of Sexual Commitment and Gametocytogenesis in Malaria Parasites. Annu. Rev. Microbiol..

[5] Bennink S., Pradel G. (2021). Vesicle Dynamics during the Egress of Malaria Gametocytes from the Red Blood Cell. Mol. Biochem. Parasitol..

[6] Trexler M., Bányai L., Patthy L. (2000). The LCCL Module. Eur. J. Biochem..

[7] Nakamura T., Tokunaga F., Morita T., Iwanaga S., Kusumoto S., Shiba T., Kobayashi T., Inoue K. (1988). Intracellular Serine-Protease Zymogen, Factor C, from Horseshoe Crab Hemocytes Its Activation by Synthetic Lipid A Analogues and Acidic Phospholipids. Eur. J. Biochem..

[8] Nakamura T., Tokunaga F., Morita T., Iwanaga S. (1988). Interaction between Lipopolysaccharide and Intracellular Serine Protease Zymogen, Factor C, from Horseshoe Crab (*Tachypleus tridentatus*) Hemocytes. J. Biochem..

[9] Muta T., Miyata T., Misumi Y., Tokunaga F., Nakamura T., Toh Y., Ikehara Y., Iwanaga S. (1991). Limulus Factor C. An Endotoxin-Sensitive Serine Protease Zymogen with a Mosaic Structure of Complement-like, Epidermal Growth Factor-like, and Lectin-like Domains. J. Biol. Chem..

[10] Robertson N.G., Skvorak A.B., Yin Y., Weremowicz S., Johnson K.R., Kovatch K.A., Battey J.F., Bieber F.R., Morton C.C. (1997). Mapping and Characterization of a Novel Cochlear Gene in Human and in Mouse: A Positional Candidate Gene for a Deafness Disorder, DFNA9. Genomics.

[11] Jung J., Yoo J.E., Choe Y.H., Park S.C., Lee H.J., Lee H.J., Noh B., Kim S.H., Kang G.-Y., Lee K.-M. (2019). Cleaved Cochlin Sequesters *Pseudomonas aeruginosa* and Activates Innate Immunity in the Inner Ear. Cell Host Microbe.

[12] Verdoodt D., Van Camp G., Ponsaerts P., Van Rompaey V. (2021). On the Pathophysiology of DFNA9: Effect of Pathogenic Variants in the COCH Gene on Inner Ear Functioning in Human and Transgenic Mice. Hear. Res..

[13] Bae S.-H., Robertson N.G., Cho H.-J., Morton C.C., Jung D.J., Baek J.-I., Choi S.-Y., Lee J., Lee K.-Y., Kim U.-K. (2014). Identification of Pathogenic Mechanisms of COCH Mutations, Abolished Cochlin Secretion, and Intracellular Aggregate Formation: Genotype-Phenotype Correlations in DFNA9 Deafness and Vestibular Disorder Formation and Retention of Dimers and Large Multimeric. Hum. Mutat..

[14] Bae S.H., Yoo J.E., Hong J.W., Park H.R., Noh B., Kim H., Kang M., Hyun Y.-M., Gee H.Y., Choi J.Y. (2021). LCCL Peptide Cleavage after Noise Exposure Exacerbates Hearing Loss and Is Associated with the Monocyte Infiltration in the Cochlea. Hear. Res..

[15] Py B.F., Gonzalez S.F., Long K., Kim M.-S., Kim Y.-A., Zhu H., Yao J., Degauque N., Villet R., Ymele-Leki P. (2013). Cochlin Produced by Follicular Dendritic Cells Promotes Antibacterial Innate Immunity. Immunity.

[16] Zhang H., Sweezey N.B., Kaplan F. (2015). LGL1 Modulates Proliferation, Apoptosis, and Migration of Human Fetal Lung Fibroblasts. Am. J. Physiol. Lung Cell. Mol. Physiol..

[17] Wang Z.-Q., Xing W.-M., Fan H.-H., Wang K.-S., Zhang H.-K., Wang Q.-W., Qi J., Yang H.-M., Yang J., Ren Y.-N. (2009). The Novel Lipopolysaccharide-Binding Protein CRISPLD2 Is a Critical Serum Protein to Regulate Endotoxin Function 1. J. Immunol..

[18] Vásárhelyi V., Trexler M., Patthy L. (2014). Both LCCL-Domains of Human CRISPLD2 Have High Affinity for Lipid A. Biochimie.

[19] Claudianos C., Dessens J.T., Trueman H.E., Arai M., Mendoza J., Butcher G.A., Crompton T., Sinden R.E. (2002). A Malaria Scavenger Receptor-like Protein Essential for Parasite Development. Mol. Microbiol..

[20] Delrieu I., Waller C.C., Mota M.M., Grainger M., Langhorne J., Holder A.A. (2002). PSLAP, a Protein with Multiple Adhesive Motifs, Is Expressed in *Plasmodium falciparum* Gametocytes. Mol. Biochem. Parasitol..

[21] Pradel G., Hayton K., Aravind L., Iyer L.M., Abrahamsen M.S., Bonawitz A., Mejia C., Templeton T.J. (2004). A Multidomain Adhesion Protein Family Expressed in *Plasmodium falciparum* Is Essential for Transmission to the Mosquito. J. Exp. Med..

[22] Templeton T.J., Iyer L.M., Anantharaman V., Enomoto S., Abrahante J.E., Subramanian G.M., Hoffman S.L., Abrahamsen M.S., Aravind L. (2004). Comparative Analysis of Apicomplexa and Genomic Diversity in Eukaryotes. Genome Res..

[23] Tosini F., Agnoli A., Mele R., Gomez Morales M.A., Pozio E. (2004). A New Modular Protein of *Cryptosporidium parvum*, with Ricin B and LCCL Domains, Expressed in the Sporozoite Invasive Stage. Mol. Biochem. Parasitol..

[24] Trueman H.E., Raine J.D., Florens L., Dessens J.T., Mendoza J., Johnson J., Waller C.C., Delrieu I., Holder A.A., Langhorne J. (2004). Functional Characterization of an LCCL-Lectin Domain Containing Protein Family in *Plasmodium berghei*. J. Parasitol..

[25] Ozubek S., Alzan H.F., Bastos R.G., Laughery J.M., Suarez C.E. (2022). Identification of CCp5 and FNPA as Novel Non-Canonical Members of the CCp Protein Family in *Babesia bovis*. Front. Vet. Sci..

[26] Wickstead B., Ersfeld K., Gull K. (2003). Repetitive Elements in Genomes of Parasitic Protozoa. Microbiol. Mol. Biol. Rev..

[27] Dessens J.T., Sinden R.E., Claudianos C. (2004). LCCL Proteins of Apicomplexan Parasites. Trends Parasitol..

[28] Pradel G., Templeton T.J., Hacker J., Dobrindt U. (2006). Genomics of Pathogenic Parasites. Pathogenomics.

[29] Rutenber E., Ready M., Robertus J.D. (1987). Structure and Evolution of Ricin B Chain. Nature.

[30] Kane W.H., Davie E.W. (1988). Blood Coagulation Factors V and VIII: Structural and Functional Similarities and Their Relationship to Hemorrhagic and Thrombotic Disorders. Blood.

[31] Baumgartner S., Hofmann K., Chiquet-Ehrismann R., Bucher P. (1998). The Discoidin Domain Family Revisited: New Members from Prokaryotes and a Homology-Based Fold Prediction. Protein Sci..

[32] Aurrecoechea C., Brestelli J., Brunk B.P., Dommer J., Fischer S., Gajria B., Gao X., Gingle A., Grant G., Harb O.S. (2009). PlasmoDB: A Functional Genomic Database for Malaria Parasites. Nucleic Acids Res..

[33] Lu S., Wang J., Chitsaz F., Derbyshire M.K., Geer R.C., Gonzales N.R., Gwadz M., Hurwitz D.I., Marchler G.H., Song J.S. (2020). CDD/SPARCLE: The Conserved Domain Database in 2020. Nucleic Acids Res..

[34] Tremp A.Z., Sharma V., Carter V., Lasonder E., Dessens J.T. (2017). LCCL Protein Complex Formation in *Plasmodium* Is Critically Dependent on LAP1. Mol. Biochem. Parasitol..

[35] Bateman A., Sandford R. (1999). The PLAT Domain: A New Piece in the PKD1 Puzzle. Curr. Biol..

[36] Hohenester E., Sasaki T., Timpl R. (1999). Crystal Structure of a Scavenger Receptor Cysteine-Rich Domain Sheds Light on an Ancient Superfamily. Nat. Struct. Biol..

[37] Resnick D., Pearson A., Krieger M. (1994). The SRCR Superfamily: A Family Reminiscent of the Ig Superfamily. Trends Biochem. Sci..

[38] Vuolteenaho R., Chow L.T., Tryggvason K. (1990). Structure of the Human Laminin Bl Chain Gene. J. Biol. Chem..

[39] Srinivasan N., White H.E., Emsley J., Wood S.P., Pepys M.B., Blundell T.L. (1994). Comparative Analyses of Pentraxins: Implications for Protomer Assembly and Ligand Binding. Structure.

[40] Gewurz H., Zhang X.-H., Lint T.F. (1995). Structure and Function of the Pentraxins. Curr. Opin. Immunol..

[41] Kuehn A., Simon N., Pradel G. (2010). Family Members Stick Together: Multi-Protein Complexes of Malaria Parasites. Med. Microbiol. Immunol..

[42] Rigden D.J., Mello L.V., Galperin M.Y. (2004). The PA14 Domain, a Conserved All-β Domain in Bacterial Toxins, Enzymes, Adhesins and Signaling Molecules. Trends Biochem. Sci..

[43] Gergely F., Karlsson C., Still I., Cowell J., Kilmartin J., Raff J.W. (2000). The TACC Domain Identifies a Family of Centrosomal Proteins That Can Interact with Microtubules. Proc. Natl. Acad. Sci. USA.

[44] Lasonder E., Ishihama Y., Andersen J.S., Vermunt A.M.W., Pain A., Sauerwein R.W., Eling W.M.C., Hall N., Waters A.P., Stunnenberg H.G. (2002). Analysis of the *Plasmodium falciparum* Proteome by High-Accuracy Mass Spectrometry. Nature.

[45] Scholz S.M., Simon N., Lavazec C., Dude M.A., Templeton T.J., Pradel G. (2008). PfCCp Proteins of *Plasmodium falciparum*: Gametocyte-Specific Expression and Role in Complement-Mediated Inhibition of Exflagellation. Int. J. Parasitol..

[46] Lasonder E., Rijpma S.R., van Schaijk B.C.L., Hoeijmakers W.A.M., Kensche P.R., Gresnigt M.S., Italiaander A., Vos M.W., Woestenenk R., Bousema T. (2016). Integrated Transcriptomic and Proteomic Analyses of *P. falciparum* Gametocytes: Molecular Insight into Sex-Specific Processes and Translational Repression. Nucleic Acids Res..

[47] Pradel G., Wagner C., Mejia C., Templeton T.J. (2006). *Plasmodium falciparum*: Co-Dependent Expression and Co-Localization of the PfCCp Multi-Adhesion Domain Proteins. Exp. Parasitol..

[48] Simon N., Scholz S.M., Moreira C.K., Templeton T.J., Kuehn A., Dude M.-A., Pradel G. (2009). Sexual Stage Adhesion Proteins Form Multi-Protein Complexes in the Malaria Parasite *Plasmodium falciparum*. J. Biol. Chem..

[49] Simon N., Kuehn A., Williamson K.C., Pradel G. (2016). Adhesion Protein Complexes of Malaria Gametocytes Assemble Following Parasite Transmission to the Mosquito. Parasitol. Int..

[50] Eksi S., Czesny B., van Gemert G.-J., Sauerwein R.W., Eling W., Williamson K.C. (2006). Malaria Transmission-Blocking Antigen, Pfs230, Mediates Human Red Blood Cell Binding to Exflagellating Male Parasites and Oocyst Production. Mol. Microbiol..

[51] Kumar N. (1987). Target Antigens of Malaria Transmission Blocking Immunity Exist as a Stable Membrane Bound Complex. Parasite Immunol..

[52] Kumar N., Wizel B. (1992). Further Characterization of Interactions between Gamete Surface Antigens of *Plasmodium falciparum*. Mol. Biochem. Parasitol..

[53] Tremp A.Z., Saeed S., Sharma V., Lasonder E., Dessens J.T. (2020). *Plasmodium berghei* LAPs Form an Extended Protein Complex That Facilitates Crystalloid Targeting and Biogenesis. J. Proteom..

[54] Bennink S., Pradel G. (2019). The Molecular Machinery of Translational Control in Malaria Parasites. Mol. Microbiol..

[55] Brooks S.R., Williamson K.C. (2000). Proteolysis of *Plasmodium falciparum* Surface Antigen, Pfs230, during Gametogenesis. Mol. Biochem. Parasitol..

[56] von Bohl A., Kuehn A., Simon N., Ngongang V.N., Spehr M., Baumeister S., Przyborski J.M., Fischer R., Pradel G. (2015). A WD40-Repeat Protein Unique to Malaria Parasites Associates with Adhesion Protein Complexes and Is Crucial for Blood Stage Progeny. Malar. J..

[57] Roling L., Flammersfeld A., Pradel G., Bennink S. (2022). The WD40-Protein Pf WLP1 Ensures Stability of the Pf CCp-Based Adhesion Protein Complex in *Plasmodium falciparum* Gametocytes. Front. Cell. Infect. Microbiol..

[58] Baum J., Richard D., Healer J., Rug M., Krnajski Z., Gilberger T.-W., Green J.L., Holder A.A., Cowman A.F. (2006). A Conserved Molecular Motor Drives Cell Invasion and Gliding Motility across Malaria Life Cycle Stages and Other Apicomplexan Parasites. J. Biol. Chem..

[59] Bargieri D.Y., Thiberge S., Tay C.L., Carey A.F., Rantz A., Hischen F., Lorthiois A., Straschil U., Singh P., Singh S. (2016). *Plasmodium* Merozoite TRAP Family Protein Is Essential for Vacuole Membrane Disruption and Gamete Egress from Erythrocytes. Cell Host Microbe.

[60] Kehrer J., Frischknecht F., Mair G.R. (2016). Proteomic Analysis of the *Plasmodium berghei* Gametocyte Egressome and Vesicular BioID of Osmiophilic Body Proteins Identifies Merozoite TRAP-like Protein (MTRAP) as an Essential Factor for Parasite Transmission. Mol. Cell. Proteom..

[61] Flieger A., Frischknecht F., Häcker G., Hornef M.W., Pradel G. (2018). Pathways of Host Cell Exit by Intracellular Pathogens. Microb. Cell.

[62] Lavazec C., Moreira C.K., Mair G.R., Waters A.P., Janse C.J., Templeton T.J. (2009). Analysis of Mutant *Plasmodium berghei* Parasites Lacking Expression of Multiple PbCCp Genes. Mol. Biochem. Parasitol..

[63] Saeed S., Carter V., Tremp A.Z., Dessens J.T. (2013). Translational Repression Controls Temporal Expression of the *Plasmodium berghei* LCCL Protein Complex. Mol. Biochem. Parasitol..

[64] Khan S.M., Franke-Fayard B., Mair G.R., Lasonder E., Janse C.J., Mann M., Waters A.P. (2005). Proteome Analysis of Separated Male and Female Gametocytes Reveals Novel Sex-Specific *Plasmodium* Biology. Cell.

[65] Raine J.D., Ecker A., Mendoza J., Tewari R., Stanway R.R., Sinden R.E. (2007). Female Inheritance of Malarial Lap Genes Is Essential for Mosquito Transmission. PLoS Pathog..

[66] Carter V., Shimizu S., Arai M., Dessens J.T. (2008). PbSR Is Synthesized in Macrogametocytes and Involved in Formation of the Malaria Crystalloids. Mol. Microbiol..

[67] Saeed S., Carter V., Tremp A.Z., Dessens J.T. (2010). *Plasmodium berghei* Crystalloids Contain Multiple LCCL Proteins. Mol. Biochem. Parasitol..

[68] Saeed S., Tremp A.Z., Dessens J.T. (2012). Conformational Co-Dependence between *Plasmodium berghei* LCCL Proteins Promotes Complex Formation and Stability. Mol. Biochem. Parasitol..

[69] Saeed S., Tremp A.Z., Dessens J.T. (2015). Biogenesis of the Crystalloid Organelle in *Plasmodium* Involves Microtubule-Dependent Vesicle Transport and Assembly. Int. J. Parasitol..

[70] Dessens J.T., Saeed S., Tremp A.Z., Carter V. (2011). Malaria Crystalloids: Specialized Structures for Parasite Transmission?. Trends Parasitol..

[71] Dessens J.T., Tremp A.Z., Saeed S. (2021). Crystalloids: Fascinating Parasite Organelles Essential for Malaria Transmission. Trends Parasitol..

[72] Saeed S., Tremp A.Z., Dessens J.T. (2018). The *Plasmodium* LAP Complex Affects Crystalloid Biogenesis and Oocyst Cell Division. Int. J. Parasitol..

[73] Guerreiro A., Deligianni E., Santos J.M., Silva P.A.G.C., Louis C., Pain A., Janse C.J., Franke-Fayard B., Carret C.K., Siden-Kiamos I. (2014). Genome-Wide RIP-Chip Analysis of Translational Repressor-Bound MRNAs in the *Plasmodium* Gametocyte. Genome Biol..

[74] Ukegbu C.V., Gomes A.R., Giorgalli M., Campos M., Bailey A.J., Besson T.R.B., Billker O., Vlachou D., Christophides G.K. (2023). Identification of Genes Required for *Plasmodium* Gametocyte-to-Sporozoite Development in the Mosquito Vector. Cell Host Microbe.

[75] Santos J.M., Duarte N., Kehrer J., Ramesar J., Avramut M.C., Koster A.J., Dessens J.T., Frischknecht F., Chevalley-Maurel S., Janse C.J. (2016). Maternally Supplied S-Acyl-Transferase Is Required for Crystalloid Organelle Formation and Transmission of the Malaria Parasite. Proc. Natl. Acad. Sci. USA.

[76] Ecker A., Bushell E.S.C., Tewari R., Sinden R.E. (2008). Reverse Genetics Screen Identifies Six Proteins Important for Malaria Development in the Mosquito. Mol. Microbiol..

[77] Saeed S., Lau C.I., Tremp A.Z., Crompton T., Dessens J.T. (2019). Dysregulated Gene Expression in Oocysts of *Plasmodium berghei* LAP Mutants. Mol. Biochem. Parasitol..

[78] Yuda M., Iwanaga S., Shigenobu S., Kato T., Kaneko I. (2010). Transcription Factor AP2-Sp and Its Target Genes in Malarial Sporozoites. Mol. Microbiol..

[79] Modrzynska K., Pfander C., Chappell L., Yu L., Suarez C., Dundas K., Gomes A.R., Goulding D., Rayner J.C., Choudhary J. (2017). A Knockout Screen of ApiAP2 Genes Reveals Networks of Interacting Transcriptional Regulators Controlling the *Plasmodium* Life Cycle. Cell Host Microbe.

[80] Ménard R., Sultan A.A., Cortes C., Altszuler R., Van Dijk M.R., Janse C.J., Waters A.P., Nussenzweig R.S., Nussenzweig V. (1997). Circumsporozoite Protein Is Required for Development of Malaria Sporozoites in Mosquitoes. Nature.

[81] Khater E.I., Sinden R.E., Dessens J.T. (2004). A Malaria Membrane Skeletal Protein Is Essential for Normal Morphogenesis, Motility, and Infectivity of Sporozoites. J. Cell Biol..

[82] Al-Khattaf F.S., Tremp A.Z., El-Houderi A., Dessens J.T. (2017). The *Plasmodium* Alveolin IMC1a Is Stabilised by Its Terminal Cysteine Motifs and Facilitates Sporozoite Morphogenesis and Infectivity in a Dose-Dependent Manner. Mol. Biochem. Parasitol..

[83] Spaccapelo R., Naitza S., Robson K.J., Crisanti A. (1997). Thrombospondin-Related Adhesive Protein (TRAP) of *Plasmodium berghei* and Parasite Motility. Lancet.

[84] Sultan A.A., Thathy V., Frevert U., Robson K.J.H., Crisanti A., Nussenzweig V., Nussenzweig R.S., Ménard R. (1997). TRAP Is Necessary for Gliding Motility and Infectivity of *Plasmodium* Sporozoites. Cell.

[85] Ishino T., Chinzei Y., Yuda M. (2005). A *Plasmodium* Sporozoite Protein with a Membrane Attack Complex Domain Is Required for Breaching the Liver Sinusoidal Cell Layer Prior to Hepatocyte Infection. Cell. Microbiol..

[86] Saeed S., Tremp A.Z., Sharma V., Lasonder E., Dessens J.T. (2020). NAD(P) Transhydrogenase Has Vital Non-Mitochondrial Functions in Malaria Parasite Transmission. EMBO Rep..

